# The primordial metabolism: an ancestral interconnection between leucine, arginine, and lysine biosynthesis

**DOI:** 10.1186/1471-2148-7-S2-S3

**Published:** 2007-08-16

**Authors:** Marco Fondi, Matteo Brilli, Giovanni Emiliani, Donatella Paffetti, Renato Fani

**Affiliations:** 1 Dipartimento di Biologia Animale e Genetica, Università di Firenze, Via Romana 17\19, Firenze, Italia; 2 Dipartimento di Scienze e Tecnologie Ambientali Forestali, Università di Firenze, Via S. Bonaventura 13, Firenze, Italia

## Abstract

**Background:**

It is generally assumed that primordial cells had small genomes with simple genes coding for enzymes able to react with a wide range of chemically related substrates, interconnecting different metabolic routes. New genes coding for enzymes with a narrowed substrate specificity arose by paralogous duplication(s) of ancestral ones and evolutionary divergence. In this way new metabolic pathways were built up by primordial cells. Useful hints to disclose the origin and evolution of ancestral metabolic routes and their interconnections can be obtained by comparing sequences of enzymes involved in the same or different metabolic routes. From this viewpoint, the lysine, arginine, and leucine biosynthetic routes represent very interesting study-models. Some of the *lys*, *arg *and *leu *genes are paralogs; this led to the suggestion that their ancestor genes might interconnect the three pathways. The aim of this work was to trace the evolutionary pathway leading to the appearance of the extant biosynthetic routes and to try to disclose the interrelationships existing between them and other pathways in the early stages of cellular evolution.

**Results:**

The comparative analysis of the genes involved in the biosynthesis of lysine, leucine, and arginine, their phylogenetic distribution and analysis revealed that the extant metabolic "grids" and their interrelationships might be the outcome of a cascade of duplication of ancestral genes that, according to the patchwork hypothesis, coded for unspecific enzymes able to react with a wide range of substrates. These genes belonged to a single common pathway in which the three biosynthetic routes were highly interconnected between them and also to methionine, threonine, and cell wall biosynthesis. A possible evolutionary model leading to the extant metabolic scenarios was also depicted.

**Conclusion:**

The whole body of data obtained in this work suggests that primordial cells synthesized leucine, lysine, and arginine through a single common metabolic pathway, whose genes underwent a set of duplication events, most of which can have predated the appearance of the last common universal ancestor of the three cell domains (Archaea, Bacteria, and Eucaryotes). The model proposes a relative timing for the appearance of the three routes and also suggests a possible evolutionary pathway for the assembly of bacterial cell-wall.

## Background

**Figure 1:**
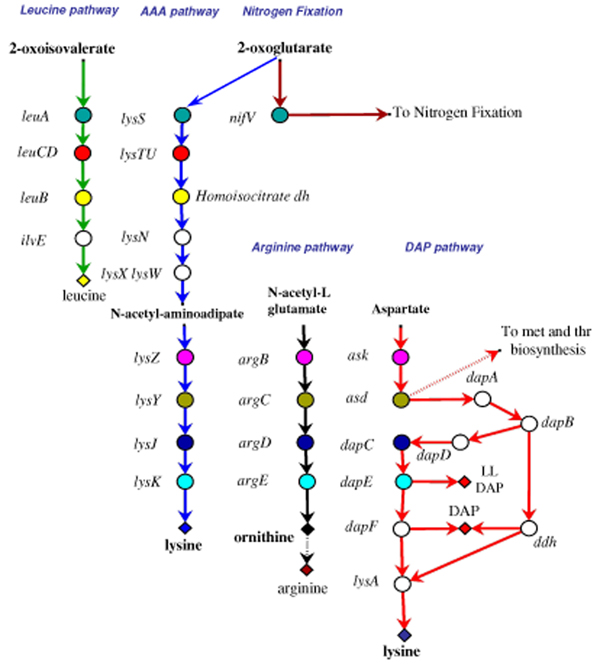
**The extant lysine, leucine, and arginine biosynthetic routes**. Evolutionary relationship between lysine, leucine, and arginine biosynthetic genes. Genes sharing the same colour and the same level are homologs. Genes coloured in white have no homolog in the metabolic routes taken into account in this work (modified from Velasco et al 2002 [19]).

It is commonly assumed that early organisms inhabited an environment rich in organic compounds spontaneously formed in the prebiotic world, an idea that is often referred to as the Oparin – Haldane theory. Those primordial organisms had no need for developing new and improved metabolic abilities since most of the required nutrients were available. However, their increasing number would have led to the depletion of essential nutrients imposing a progressively stronger selective pressure that, in turn, favoured those (micro)organisms that have become able to synthesize the nutrients whose concentration was decreasing in the primordial soup. Thus, the origin and the evolution of basic biosynthetic pathways represented a crucial step in cellular evolution, since it rendered the primordial cells less dependent on the external source of nutrients. But how did these metabolic pathways emerge and evolve? Several theories have been proposed to explain how the metabolic routes have been assembled (see [1] and reference therein). Even though it is possible that different processes might have been responsible for the build up of metabolic routes, a large body of data concerning both sequence comparison and experimental data on enzymes substrate specificity strongly supports one of them, that is the *patchwork assembly *theory [2]. According to this idea, metabolic pathways might have been originated through the recruitment of primitive enzymes that could react with a wide range of chemically related substrates. Such relatively slow, unspecific enzymes may have enabled primitive cells containing small genomes to overcome their limited coding capabilities. Paralogous gene duplication event(s) followed by evolutionary divergence might have permitted the appearance of enzymes with an increased and narrowed specificity and/or the functional diversification. In this way, an ancestral enzyme belonging to a given metabolic route was "recruited" to serve a single or other (novel) pathways. The importance of gene duplication in the course of evolution of genomes and metabolic pathways is well established [3]: the production of two (or more) copies of a DNA sequence leads to an increase of genome size, and it also allows the (rapid) diversification of enzymes, providing material for the invention of new enzymatic properties and complex regulatory and developmental patterns. Therefore, gene duplication should have had a deep impact on primordial metabolism. Indeed, it is quite possible that the biochemical flexibility of the ancestral enzymes might result in an extreme interconnection among different metabolic routes. Hence, the duplication event(s) followed by evolutionary divergence, allowing gaining of novel metabolic capabilities, permitted the new metabolic pathways to be less branched and interconnected to each other. How can this issue be studied? Useful hints to disclose the origin and evolution of metabolic pathways and the possible ancestral interrelationships between different routes may be obtained by comparing the sequence and the structure of genes (and/or the products they code for) of the same and different routes from (micro)organisms belonging to the three cells domains (Archaea, Bacteria and Eucarya). In this context the lysine, arginine, and leucine biosynthetic pathways represent interesting study-models. One of the main reasons is the existence of two well-distinct routes that have been characterized for the anabolism of lysine, that is the α-aminoadipate (AAA) pathway and the diaminopimelate (DAP) one [4-6] (Figure 1). The first one starts from 2-oxoglutarate and leads to lysine, through nine steps, one of which (catalyzed by LysN) is responsible for the formation of α-aminoadipate. Up to now, genes belonging to this pathway have been found in a limited number of (micro)organisms, such as the Bacteria *Thermus thermophilus *and *Deinococcus radiodurans *[7-9] and the Archaea *Pyrococcus *[8], *Thermoproteus *[10], and (probably) *Sulfolobus *[10,11]. A distinct variant of the AAA pathway has been disclosed in higher Fungi [6,12-14] and in euglenoids [15,16]. The alternative route leading to lysine, referred to as the DAP pathway, involves nine enzymatic reactions and produces lysine starting from L-aspartate. The DAP pathway also plays a central role in cell-wall biosynthesis of gram-negative bacteria, since meso-diaminopimelate is an essential precursor in the biosynthesis of peptidoglycan [17,18]. Genes involved in the DAP pathway are widespread in both Prokaryotes and Eucaryotes [19,20]. Interestingly, AAA and DAP pathways are evolutionary linked to leucine and arginine biosynthesis. The relationship existing between genes belonging to these biosynthetic routes has been previously analyzed ([19] and reference therein) and is schematically represented in Figure 1. The products of the four genes involved in the DAP pathway (*ask*, *asd*, *dapC*, and *dapE*) are evolutionary related to arginine biosynthetic enzymes encoded by *argB*, *argC*, *argD *and *argE*, respectively [19]. Some of the enzymes involved in the AAA pathway share a high degree of sequence similarity with enzymes belonging both to leucine and arginine biosynthetic routes [8,19] since the first four enzymes of the *Thermus*-like AAA biosynthetic pathway (LysS, LysT, LysU, and Homoisocitrate dehydrogenase) are homologous to the corresponding enzymes of leucine biosynthesis (LeuA, LeuC, LeuD, LeuB) [8,19,21]. Moreover LysZ, LysY, LysJ and LysK are homologous to ArgB, ArgC, ArgD, and ArgE, respectively [19,22]. The high degree of sequence similarity shared by these enzymes led to the suggestion that: i) the assembly of both the DAP and AAA routes might be explained as the outcome of a series of gene duplication events followed by specialization [19], ii) the DAP route should represent the ancestral pathway leading to lysine and, iii) the AAA pathway should be a more recent invention of evolution[19].

However, in spite of the available data, no evolutionary model explaining the extant scenario has been proposed. The aim of this work was to try to trace the evolutionary history of the three metabolic pathways and to shed some light on the ancestral route(s) and interrelationships existing between them and (eventually) with other metabolic routes. To this purpose, a comparative analysis of the extant leucine, arginine, and lysine metabolic pathways from (micro)organisms whose genome has been completely sequenced was carried out.

## Results and discussion

### Distribution of leucine, arginine and lysine biosynthetic genes

**Figure 2:**
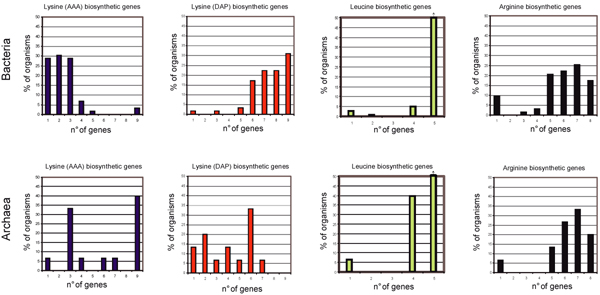
**Distribution of lysine, leucine, arginine biosynthetic genes**. Histogram showing the number of lysine, leucine, and arginine biosynthetic genes possessed by Bacteria and Archaea.

The amino acid sequence of each of the *E. coli *lysine (DAP), leucine, and *T. thermophilus *lysine (AAA) biosynthetic enzymes, was used as a query to probe the completely sequenced genomes database of KEGG (Kyoto Encyclopaedia of Genes and Genomes) consisting of 29 Archaea, 423 Bacteria and 35 Eucaryotes. The bidirectional best-hit (BBH) criterion (see Methods) was used to retrieve orthologous sequences. Data obtained, schematically reported in Figure 2 and representative of a dataset of 68 bacterial and 15 archaeal genomes, revealed that:

i) Lysine (AAA) biosynthetic genes are very rarely represented in Bacteria, in that just two organisms harbour the complete set of AAA biosynthetic genes, i.e. *T. thermophilus *and *D. radiodurans*. No other bacterium possesses the complete set of enzymes required to synthesize lysine via the AAA route. Six Archaea display a complete set of lysine (AAA) biosynthetic genes, namely *Pyrococcus *and *Sulfolobus *strains, and *Thermococcus kodakaraensis*.

ii) Lysine (DAP) biosynthetic genes are widespread among Bacteria. Among them, 18 (micro)organisms possess a complete set of genes (9) for lysine biosynthesis through the DAP route. The small number of lysine (DAP) biosynthetic genes in some bacterial strains is very likely due to the absence of the corresponding metabolic route, which, in turn, is related to the parasitic lifestyle of these organisms. Such a lifestyle may allow these bacteria to acquire essential compounds directly from the metabolic activities of their host and the adaptation to this environmental condition might have caused the loss of entire metabolic routes or parts thereof.

Although some Archaea are known to synthesize lysine through the DAP pathway [23-28], it is not still completely clear which of the possible variants they use. Nonetheless, no archaeon possesses a number of genes compatible with the number required by the succinylasic variant. When the *ddh *sequence of *Corynebacterium glutamicum*, whose product is involved in the dehydrogenasic variant of lysine (DAP) pathway, was used in a BLAST probing, the only *ddh *archaeal homolog sequence was found in *Archaeoglobus fulgidus *genome (data not shown).

iii) The complete set of leucine biosynthetic genes is present in most of Bacteria and Archaea that possess either all the five leucine biosynthetic genes or four of them (lacking the last gene of leucine biosynthetic route, *ilvE*). This different distribution of *ilvE*, had already been observed by Velasco *et al*. [19].

iv) Arginine biosynthetic genes are widespread among Archaea and Bacteria. Even though a complete set is found only in a restricted number of organisms, most of them possess more than half (from five up to eight) of the enzymes required for the biosynthesis of ornithine and arginine, confirming the previous observation that they are synthesized through *N*-acetylated intermediates both in Bacteria and in Archaea [29].

### Structure of leucine, arginine, and lysine biosynthetic pathways

**Figure 3:**
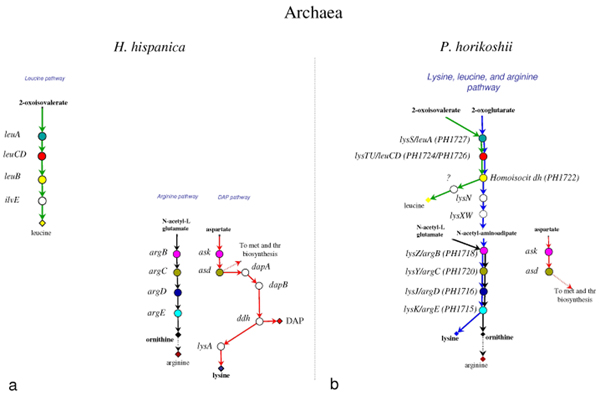
**Structure of lysine, leucine, and arginine biosynthetic routes in the Archaea *H. hispanica *and *P. horikoshii***. The lysine, leucine, and arginine biosynthetic routes in *H. hispanica ***(a) **and in *P. horikoshii ***(b) **(see references in the text). Genes sharing the same colour and the same level are homologs. Genes coloured in white have no homolog in the metabolic routes studied in this work.

**Figure 4:**
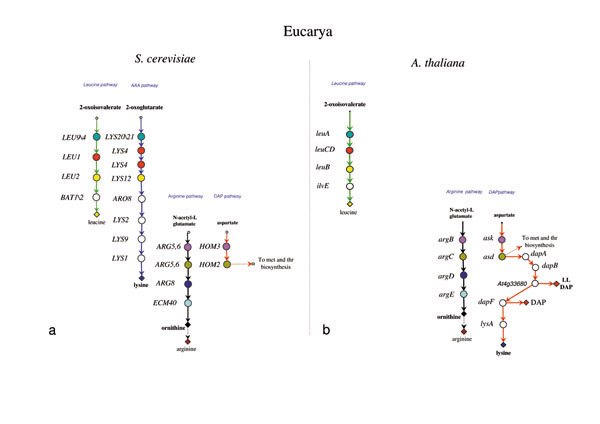
**Structure of lysine, leucine, and arginine biosynthetic routes the Eucarya *S. cerevisiae *and *A. thaliana*. **The lysine, leucine, and arginine biosynthetic routes in *S. cerevisiae ***(a) **and in *A. thaliana ***(b) **(see references in the text). Genes sharing the same colour and the same level are homologs. Genes coloured in white have no homolog in the metabolic routes studied in this work.

**Figure 5:**
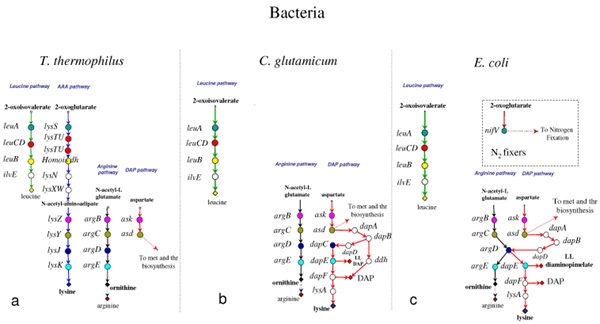
**Structure of lysine, leucine, and arginine biosynthetic routes in some Bacteria**. The lysine, leucine, and arginine biosynthetic routes in *T. thermophilus ***(a)**, in *C. glutamicum ***(b)**, and in *E. coli ***(c) **(see references in the text). Genes sharing the same colour on the same level are homologs. Genes coloured in white have no homologs in the metabolic routes studied in this work.

A comparative analysis of the lysine, leucine, and arginine biosynthetic routes of completely sequenced organisms belonging to the three cellular domains (Eucarya, Bacteria, and Archaea) was carried out. Data obtained for some representative organisms belonging to the three cellular domains are shown in Figures 3, 4, and 5 and can be summarised as reported below.

In most of archaeal, bacterial, and eukaryotic (micro)organisms each of the three amino acid is synthesized through a specific metabolic route and each enzyme catalyzes a single step of the metabolic pathway it belongs to. The only exception is represented by the archaeon *Pyrococcus horikoshii *(see below); moreover a bifunctional enzyme, coded for by *argD*, interconnecting arginine and lysine biosyntheses was identified and characterized in *Escherichia coli *(see below).

In all of the organisms analyzed, leucine is synthesized through the typical biosynthetic pathway, consisting of the enzymatic steps catalyzed by 3-isopropylmalate synthase, 2-isopropylmalate isomerase, 3-isopropylmalate dehydrogenase, and α-ketoisocaproate transaminase, respectively. The four enzymes are coded for by the procaryotic *leuA*, *leuCD*, *leuB*, and *ilvE *genes, or by the eucaryotic *LEU9\4*, *LEU1*, *LEU2*, and *BAT1\2 *(Figures 3, 4, 5).

Similarly, arginine biosynthesis proceeds through the same steps in all the organisms taken into account (Figures 3, 4, 5).

The lysine biosynthesis is much more intriguing; the amino acid can be synthesized through either the AAA or the DAP pathway (see Background). The AAA pathway was found in *T. thermophilus *and *D. radiodurans*, and a modified version of it was found in *S. cerevisiae *(Figure 4a and Figure 5a). The first four metabolic steps are shared by the two organisms and are evolutionarily related to the corresponding ones of leucine biosynthesis [8,19]. The second moiety of these two AAA versions is different. In *T. thermophilus *lysine biosynthesis is achieved by the products of *lysZ*, *Y*, *J*, and *K*, whose sequences are homologous to *argB*, *C*, *D*, and *E*, respectively, whereas in *S. cerevisiae *lysine biosynthesis is completed by *LYS2*, *LYS9*, *LYS1*, that have no homolog among the genes of the biosynthetic pathways we took into account.

In the other Bacteria and in some Archaea, lysine is synthesized through the DAP pathway. Even in this case, several alternatives are possible. In the γ-proteobacterium *E. coli *(Figure 4c) the product of DapB is converted in LL-meso-diaminopimelate through four sequential enzymatic steps, known as the succinylate variant, and performed by DapD, ArgD, DapE, and DapF. Moreover, the enzyme coded for by *argD *exhibits both N-acetyl-ornithine and N-succinyl-L,L-diaminopimelate aminotransferase activities, with a very similar catalytic efficiency and identical kinetic mechanism, suggesting that this enzyme can play a role in both lysine and arginine biosynthesis [30]. In *C. glutamicum *(Figure 4b) another version of the pathway has been disclosed: tetrahydrodipicolinate can be converted into meso-diaminopimelate by the activity of diaminopimelate dehydrogenase (*ddh*) in a single metabolic step. It is highly likely that this catalytic step is present also in the DAP pathway of the archaeon *Haloarcula hispanica *(Figure 3a) [23]. Interestingly, in *C. glutamicum *two distinct enzymes (ArgD and DapC) can perform the reaction that in *E. coli *is carried out by a single one. Thus, the enzymes encoded by *argD *and *dapC *in *C. glutamicum *can be considered specific for the biosynthetic routes of arginine and lysine, respectively.

*Arabidopsis thaliana *is known to synthesize lysine through the DAP pathway [15,31]. A DAP variant has been recently identified in this plant that utilizes a novel transaminase (LL-aminoadipate aminotransferase, the product of ORF *At4g33680*) that specifically catalyzes the conversion of tetrahydrodipicolinate to LL-diaminopimelate, a reaction requiring three enzymes in the DAP-pathway variant found in *E. coli *[32].

The archaeon *P. horikoshii *represents the main exception. In fact, in this organism just one pathway for the biosynthesis of all these three amino acids has been identified so far (Figure 3b). On the basis of sequence comparison and phylogenetic analyses, it has been suggested [9] that ORF PH1722, PH1724, PH1726, and PH1727 (see Figure 3b) from *P. horikoshii *might be involved in leucine biosynthesis as well as in the AAA variant of lysine biosynthesis, and that ORF PH1720, PH1718, PH1716, and PH1715 (Figure 3b) might be involved in the biosynthesis of both lysine (through the AAA one) and arginine. Even though it cannot be ruled *a priori *out the possibility that other uncharacterized routes for the biosynthesis of these amino acids may exist [33], it has been suggested that *P. horikoshii *possesses a unique amino acid biosynthetic system by which several amino acids are synthesized by a limited number of enzymes with broad substrate specificity [8].

### A hypothetical ancestral pathway for lysine, leucine, and arginine

Data shown in Figures 3, 4 and 5 reveal that some steps of lysine, leucine, and arginine biosynthetic pathways are strongly conserved among the organisms we have taken into account. These steps are the first three of leucine route (performed by LeuA, LeuCD, and LeuB), the central ones of the arginine route (performed by ArgB, ArgC, ArgD, and ArgE), and the first two of lysine (DAP) route (performed by Ask and Asd). Moreover, these enzymes, share a significant degree of sequence similarity with some of those involved in the AAA lysine biosynthesis [19]. As shown in Figure 1, *leuA*, *leuCD*, and *leuB *appeared to be paralogous to *lysS*, *lysTU*, and homoisocitrate dehydrogenase coding gene, respectively. A similar paralogy exists between *argB*, *ask*, and *lysZ*, as well as among *argC*, *asd*, and *lysY*. Velasco *et al*. [19] suggested that all of them are the outcome of one or more duplication events of an ancestral set of genes. According to the patchwork hypothesis [2], these ancestral genes might have encoded enzymes possessing broad substrate specificity and have been involved in different metabolic pathways.

**Figure 6:**
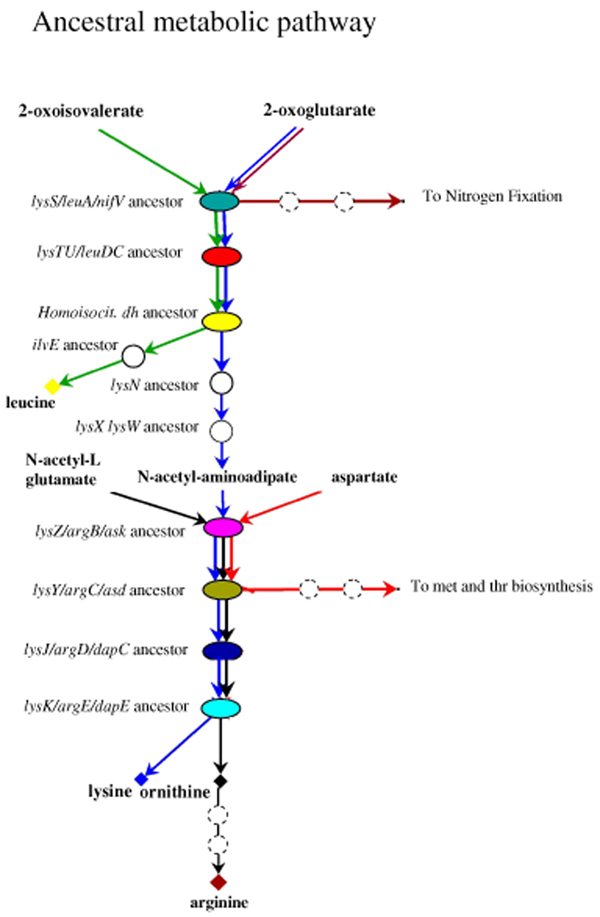
**A hypothetical ancestral common route for lysine, leucine, and arginine**. The ancestral metabolic pathway, constituted by a restricted, unspecific set of enzymes, able to synthesize leucine, lysine, ornithine, and LL-diaminopimelate. Coloured genes are related to the extant lysine, leucine, and arginine biosynthetic ones. Dashed circles indicate more than one metabolic step. Genes coloured in white have no homologs in the metabolic routes studied in this work.

On the basis of the analysis of phylogenetic distribution, Velasco *et al*. [19] also proposed that the DAP variant of lysine biosynthesis appeared earlier than the AAA one. However, a different scenario can be proposed. The model that we describe here predicts the existence of an ancestral pathway consisting of a set of genes, some of which coding for unspecific enzymes able to react with a wide range of chemically related substrates. This metabolic pathway interconnected lysine, leucine, arginine and also methionine and threonine biosyntheses and nitrogen fixation (Figure 6). According to the model, the first step of the primordial route was catalyzed by the ancestor of the extant isopropylmalate synthase (IPMase, LeuA) and homocitrate synthase (HCase, NifV), that are involved in leucine biosynthesis and nitrogen fixation, respectively. The paralogy existing between their coding genes has not been analyzed in detail up to now, but its description is beyond the scope of the present work. Moreover, the first three steps of this ancestral biosynthetic pathway might have included the reactions that, in the extant organisms, are separately accomplished by the enzymes of leucine and lysine-AAA pathways. Moreover, the last four steps, involving the ancestral copies of the extant ArgB, C, D, and LysZ, Y, J, K, may have been able to recognize different substrates and to catalyze their conversion into ornithine, a fundamental intermediary in arginine biosynthesis.

Even though these ancestral enzymes may have been the backbone of the hypothetical common route, some others might have been necessary to complete the biosynthesis of all the corresponding products. An ancestor of the extant IlvE might have catalyzed the conversion of 2-oxoisocaproate into leucine, the final step of leucine biosynthesis, whereas an unspecific aminotransferase may have accomplished the transamination of α-aminoadipate, the extant role of LysN.

Moreover, an ancestral copy of LysX, which is evolutionarily related to *E. coli *RimK [34], was probably involved in the modification of α-aminoadipate to N-acetyl-α-aminoadipate, releasing the substrate of LysZ/ArgB/Ask ancestor. As shown in Figure 5, the model proposed does not contemplate the biosynthesis of LL-diaminopimelate in primordial cells, implying that the AAA pathway predated the appearance of the DAP pathway.

In conclusion, ancestral organisms may have been able to synthesize lysine, leucine and ornithine using just one pathway, consisting of a small number of enzymes endowed with a broad substrate specificity, permitting a clear interconnection of different metabolic routes.

### A model for the evolution of lysine, leucine, and arginine metabolic routes

On the basis of the structure and the relationships existing in the extant biosynthetic routes leading to lysine, leucine, or arginine we depict a possible evolutionary model that might explain the (complex) extant scenario.

**Figure 7:**
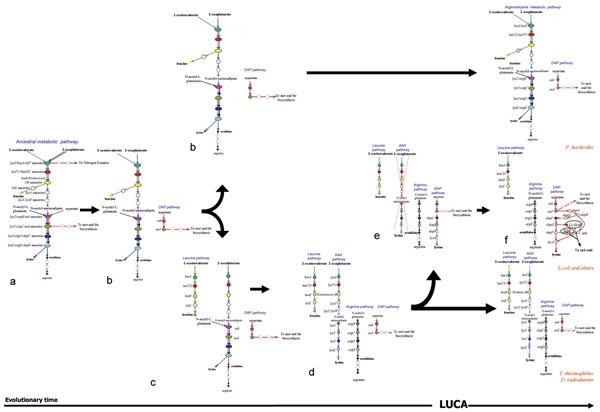
**Evolutionary model for the assembly of lysine, leucine, and arginine biosynthetic pathways**. Evolutionary model proposed to explain the evolution and the assembly of lysine, leucine, and arginine biosynthetic pathways starting from the hypothetical common route. Dashed circles indicates more than one metabolic step.

The model predicts the existence of the above described ancestral pathway (Figures 6 and 7a) that appears to be very similar to that responsible for the biosynthesis of lysine, leucine, and ornithine in *P. horikoshii *(Figure 3b and 6). It has been proposed [8] that *P. horikoshii *might have developed unique amino acid biosynthetic systems in which several amino acids are synthesized by a limited number of enzymes with broad substrate specificity and that these enzymes might be the ancestors of the extant biosynthetic ones of lysine, leucine, and arginine.

If the idea of an ancestral common pathway for the biosynthesis of lysine, leucine and arginine is correct, this raises the question of how did the extant routes originate. Besides, which was the timing of their appearance? Here we propose a model that is in agreement with the Nishida's idea and predicts that the extant biosynthetic routes might have emerged after a set of duplication events involving the genes of the ancestral common pathway (Figure 7). According to the model, the first step (or one of the first steps) might have been the duplication of the genes encoding the enzymes catalysing the sixth and seventh steps of the route common to lysine and ornithine. These events gave raise to the ancestors of *ask *and *asd *genes and to the overall metabolic "grid" that has been found in *P. horikoshii *(Figures 3b and 7b). This idea is also supported by the phylogenetic analysis (see next section).

Later on, the duplication of the genes encoding the first three steps of the ancestral pathway and the further evolutionary divergence of the new copies might have originated the ancestor of *leuA*, *leuCD*, and *leuB*, which coded for enzymes with a more narrow substrate specificity, rendering the leucine biosynthesis independent from the ancestral common pathway (Figure 7c). The central steps of arginine biosynthesis, catalyzed by ArgB, ArgC, ArgD and ArgE, might have arisen from the duplication and further evolutionary divergence of the corresponding ancestral aspecific genes (Figure 7d). In this way the four metabolic routes leading to leucine, lysine, arginine, and methionine\threonine, respectively, became one independent from each other. This metabolic scheme corresponds to that found in *T. thermophilus *and *D. radiodurans*.

A further duplication of the bottom genes of the ancestral pathway led to the appearance of DapC and DapE; during this step the branch of the "DAP pathway" leading to lysine was completely assembled (Figure 7e) with the recruitment of *dapC *and *dapE *(that are homolog to the extant *lysJ/argD *and *lysZ/argB*, respectively), *dapF*, and *lysA*. On the basis of the available data, it is not still possible to discern between the possibility that the product of the reaction catalyzed by Asd directly interacts with DapC, or if other enzymes (possibly the ancestor of the extant *dapA*, *dapB*, and *dapD*) were required to complete the DAP route. However, it is possible that once the assembly of the lysine-branch of the DAP pathway was completed the primordial cells possessed two alternative ways to synthesize lysine. If the model we propose here is correct, it is possible that, in this context, the entire AAA pathway became superfluous. Hence, the fourth step (Figure 7e) of the model predicts that, after the completion of the DAP pathway to lysine, genes belonging to the AAA pathway were progressively lost; therefore those organisms lacking the DAP pathway, maintained the ability to synthesize lysine through the AAA route. The final step of the model (Figure 7f) predicts that lysine (DAP) may have been completely assembled, with the appearance of all the extant variants (deacetylasic, desuccynilasic, and dehydrogenasic), leading to the metabolic patterns found in *E. coli *and in many other microorganisms. The development of the DAP pathway might allow bacterial cells to acquire the possibility to insert LL-diaminopimelate and meso-diaminopimelate, that are both intermediaries of the DAP route, into their cell wall.

In our opinion, the duplication events from Figure 7a to Figure 7e would have predated the appearance of the Last Universal Common Ancestor (LUCA), which, according to Woese [35], would have been a complex community of progenotes, highly dynamic from a genomic viewpoint, a mix of heterogeneous primordial cells with different metabolic abilities that could be easily exchanged between the different entities of the community. According to this idea, cells with different metabolic grids such as those represented in Figure 7b and 7d might have co-existed in the "LUCA community". The model also predicts, according to Cavalier-Smith [36], that if the primordial cells were surrounded by a cell wall containing peptidoglycan, ornithine should have been one of the first, if not the very first, component of peptidoglycan (see *Conclusions*).

### Phylogenetic analysis

According to the model proposed, the metabolic pathway leading to methionine and threonine diverged from the ancestral pathway before lysine, leucine, and arginine biosynthetic ones. However, (at least) another plausible scenario can be depicted, involving the previous appearance of the leucine pathway and the further assembly of the others biosynthetic routes. In other words, is it possible to establish the relative timing of the separation of the extant three pairs of genes involved in lysine, arginine, threonine, and methionine biosynthesis, that is *lysZ *and *lysY, argB *and *argC*, and *ask *and *asd*?

**Figure 8:**
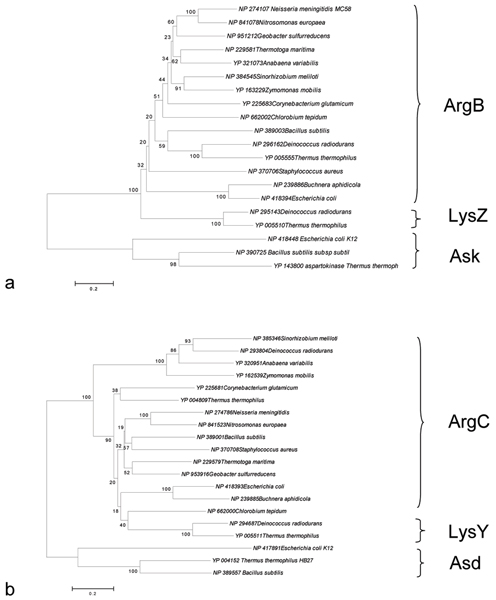
**Phylogenetic trees**. Phylogenetic trees (Neighbor Joining, 2250 Bootstrap Replicates, Complete Deletion, Poisson Correction) constructed with the sequences of LysZ, ArgB, Ask **(a) **and LysY, ArgC, Asd **(b)**.

Useful hints concerning this issue can be obtained by a phylogenetic analysis of the two paralogous triads (*LysZ*, *ArgB*, *Ask*, and *LysY, ArgC*, and *Asd*). To this purpose a dataset of all the retrieved LysZ, ArgB, Ask and, LysY, ArgC, and Asd amino acid sequences was aligned using the program ClustalW [37] and the multialignments obtained used to draw the phylogenetic trees shown in Figure 8. This analysis revealed that:

1. Ask sequences form a unique cluster, which is clearly separated from the other one containing all the ArgB and LysZ sequences involved in arginine and lysine (AAA) biosynthesis, respectively (Figure 8a).

2. Asd sequences form a unique cluster separated from ArgC and LysY sequences, involved in arginine and lysine biosynthesis, respectively (Figure 8b).

Hence, the topology of the tree in Figure 8a suggests that ArgB and LysZ sequences share a degree of sequence similarity higher than that exhibited with Ask, respectively. Similarly, the tree shown in Figure 8b, revealed that ArgC and LysY sequences share a degree of similarity higher than that exhibited with Asd. The overall body of phylogenetic data suggests that the first duplication event(s) involving the ancestral common genes might have originated the ancestor of the extant *ask *and *asd *genes; hence, *argB *and *argC *might be the outcome of a later duplication event of their corresponding ancestral sequences.

Thus, in our opinion, the model proposed in the previous section appeared to be in agreement with both phylogenetic analyses and the metabolic schemes shown in Figures 2, 3, 4.

## Conclusion

In this work a likely model for the evolution of genes involved in the biosynthesis of lysine, leucine, and arginine is depicted. The model proposed is based on the analysis of the structure of these biosynthetic routes and the phylogenetic distribution of their genes. The phylogenetic analysis performed allowed us also to determine a possible relative timing of the appearance of genes that are involved in the extant lysine (DAP) and arginine biosynthetic routes. This analysis gave a strong support to the hypothesis that extensive gene duplication events played a key role in shaping the extant biosynthetic routes of lysine, leucine and arginine. According to the model proposed in this work a common metabolic pathway for the biosynthesis of these three amino acids predated the appearance of the last universal common ancestor. This ancestral metabolic route was probably composed of a set of unspecific enzymes able to react with chemically related substrates interconnecting different biosynthetic routes (Figure 6). The occurrence of multiple gene duplication events (Figure 7) would have led to the appearance of specific metabolic pathways responsible for the biosynthesis of each amino acid. The evolutionary history of lysine, leucine, and arginine biosynthetic routes strongly supports the hypothesis on the origin and evolution of metabolic pathways proposed by Jensen [2], strengthening the idea that the gene duplication and the recruitment of genes encoding enzymes with a broad substrate specificity played a key role in the assembly of primitive metabolic routes.

Hence, starting from a metabolic network whose (highly interconnected) nodes represent unspecific enzymes (Figure 6), novel metabolic networks have emerged, consisting of highly specialized (and less interconnected) enzymes (Figure 1). In this way, ancestral enzymes belonging to a given metabolic route, have been "recruited" to serve a single or other (novel) pathways.

One of the main consequences of this evolutionary pathway, i.e. gene duplication followed by evolutionary divergence, is the separation of metabolic routes that were originally fused in a single, common one. The biological significance of this cascade of duplication events might rely on both (i) the appearance and refinement of regulatory mechanisms specific to each novel metabolic pathway and on (ii) the increased robustness acquired by the whole metabolic network, i.e. its ability to respond to environmental changes or to "knock-out" mutations falling within biosynthetic genes (while maintaining unchanged biosynthetic activity). In fact, the removal of one of the multifunctional enzymes belonging to the ancestral common pathway of lysine, leucine and arginine (Figure 6) would lead to auxotrophy to all of the three amino acids. On the contrary, mutations silencing one of the extant, specific biosynthetic genes may generate auxotrophy only for the amino acid whose biosynthetic route has been interrupted, whereas all the others may still be synthesized. In this latter case, the presence of a paralogous copy may still account for the reaction catalyzed by the product of the silenced gene, permitting the biosynthesis of the corresponding amino acid and avoiding the occurrence of any other auxotrophy. In this context the *dapC *and *argD *genes (Figure 14b, 4c) represent a paradigmatic example. In *E. coli *(Figure 4c) the *argD *product exhibits both N-acetyl-ornithine and N-succinyl-L,L-diaminopimelate aminotransferase activities [30] whereas in *C. glutamicum *two distinct enzymes, DapC and ArgDrespectively, perform these reactions [38]. Although the genetic inactivation of *E. coli argD *and the consequences of this disruption are still under investigation, it is likely that it may generate auxotrophy to both diaminopimelate (and lysine) and L-arginine [30]. On the contrary, the simultaneous deletion of the *dapC *gene and *ddh *in *C. glutamicum *does not generate auxotrophy to lysine, suggesting that the product of *argD *may substitute the DapC function [38]. Since the simultaneous deletion of *dapC*, *ddh *and *argD *genes from *C. glutamicum *does not affect its growth, the existence of another aminotransferase able to substitute ArgD and DapC, has been invoked [38]. This, in turn, is in agreement with the well-established low substrate specificity of aminotransferases [39], that may allow the enzymes carrying this activity to serve several different metabolic pathways.

On the basis of gene distribution analysis, Velasco *et al*. [19] proposed that the DAP pathway appeared earlier than the AAA one. The model we have proposed is in disagreement with this view, in that it suggests that the AAA pathway is the oldest one and that both pathways might have been simultaneously (and transiently) present in the "LUCA community". Since most bacteria, with the only exception of *T. thermophilus *and *D. radiodurans *(TD group), lack the AAA pathway, we suggest that, during the separation of Archaea from Bacteria, the latter may have lost the AAA pathway, maintaining the DAP pathway for the biosynthesis of lysine, LL-diaminopimelate, and meso-diaminopimelate (Figure 7e). The presence of the AAA pathway in the TD group might be the result of an event of horizontal gene transfer between an ancestor of *P. horikoshii *and the ancestor of the (micro)organisms belonging to the TD group as suggested by Nishida [9]. Alternatively, *T. thermophilus *and *D. radiodurans *have maintained the AAA pathway. The model proposed fits with the metabolic schemes shown in Figures 2, 3, 4 and the timing of some duplication events is supported by the phylogenetic analysis of triads of paralogous genes.

The distribution of the AAA and the DAP pathway in Archaea defies a simple explanation since all currently known pathways for lysine biosynthesis in Bacteria and Eucarya exist also in the domain of Archaea [23]. Hence, in the case of Archaea, a lineage specific maintenance of the DAP or the AAA pathway for lysine biosynthesis seems to be the most reliable hypothesis.

Lastly, on the basis of the scenario depicted in this work, the evolutionary history of lysine, leucine, and arginine biosynthetic routes might supply useful hints to disclose the origin and the assembly of bacterial cell wall. In the extant bacteria, the rigidity of the cell wall is due to a huge macromolecule containing acylated amino sugars and three to six different amino acids. This heteropolymer, the peptidoglycan, is built out of glycan strands cross linked through short peptides. In contrast to the uniform structure of the glycan, the peptide moiety reveals considerable variations. The greatest variation occurs at position 3 of the peptide moiety, where usually a diamino acid is found. The most widely distributed diamino acid is meso-diaminopimelate. It is present in gram-negative bacteria and in many other (micro)organisms, such as some species of bacilli, clostridia, lactobacilli, corynebacteria, propionibacteria, actinomycetales, myxobacteriales, rickettsiae, and cyanobacteria. Some studies have shown that the L-asymmetric carbon of meso-diaminopimelate is bound to the peptide subunit (see [40] and references therein). Studies on the amino acid composition and sequence of the peptidoglycans of different gram-negative bacteria have also shown that there is no great variation within this group. The peptidoglycan of these bacteria contains mainly meso-diaminopimelate, although D-glutamate and L-alanine can replace it.

The gram-positive bacteria reveal, contrary to the gram-negative organisms, a great variability in the composition and structural arrangement of their peptidoglycan since it can alternatively contain, at position 3, L-lysine, L-ornithine, D-glutamate, and LL-diaminopimelate.

Lastly, archaeal cells possess a fundamentally different type of peptidoglycan. Its glycan moiety contains L-talosaminuronic acid instead of muramic acid, and its peptide moiety lacks D-amino acids, but it is present L-lysine (see [40] and references therein).

As expected by the lack of DAP pathway, all those bacterial (micro)organisms biosynthesizing lysine through the AAA pathway (*T. thermophilus*, *D. radiodurans*) do not have diaminopimelic acid within their cell wall [41,42]. In these microorganisms diaminopimelic acid is replaced by ornithine. In addition to this, LL-diaminopimelate and meso-diaminopimelate, intermediaries of the DAP pathway (Figure 1), are compounds that can be found in the cell wall of extant Bacteria [40], whereas they are absent in the archaeal cell wall of Archaea [43]. Hence we suggest that the maintenance of the DAP pathway for the biosynthesis of lysine, at least in Bacteria, might be correlated with the appearance of the extant structure of their cell wall. The ability of inserting these molecules in the cell wall, might be an "invention" of Bacteria. In our opinion, the primordial cells had ornithine as a component of peptidoglycan. This is in agreement with the recent suggestion that the presence of ornithine within the cell wall might be an ancestral feature [36] whose biosynthesis, in the primordial organisms, might have occurred through the previously proposed common route (Figure 6).

This evolutionary step corresponds to the last one that is depicted in the evolutionary model that we have previously proposed (Figure 7f). This view might support the idea that the insertion of the meso-diaminopimelate and, probably, the appearance of the fully assembled DAP pathway might be a metabolic invention of Bacteria.

## Methods

### Sequence retrieval

Amino acid sequences were retrieved from GenBank and KEGG databases and were used to build a local database for BLASTp [44] probing that was performed using default parameters. Orthologs identification was achieved according to the bidirectional best-hit (BBH) criterion [45,46]. The relationship between gene *x *in genome A and gene *y *in genome B is called best-best hit when *x *is the best hit of query *y *against all genes in A and *vice versa*, and it is often used as an operational definition of ortholog [45,47].

### Sequence alignment

The ClustalW [37] program in the BioEdit [48] package was used to perform pairwise and multiple amino acid sequences alignments.

### Phylogenetic analysis

Phylogenetic trees were obtained with Mega 3 software [49] using the Neighbor-Joining (NJ) and the Minimum Evolution (ME) methods.

## Competing interests

The authors declare that they have no competing interests.

## Authors' contributions

All authors equally contributed to the preparation of the final version of the manuscript; MF performed the analyses during its PhD thesis under the supervision of Prof. RF.
